# Spatially Resolved Defect Characterization and Fidelity Assessment for Complex and Arbitrary Irregular 3D Printing Based on 3D P-OCT and GCode

**DOI:** 10.3390/s24113636

**Published:** 2024-06-04

**Authors:** Bowen Fan, Shanshan Yang, Ling Wang, Mingen Xu

**Affiliations:** 1 School of Automation, Hangzhou Dianzi University, Hangzhou 310018, China; 212060352@hdu.edu.cn (B.F.); xumingen@hdu.edu.cn (M.X.); 2 Zhejiang Provincial Key Laboratory of Medical Information and Biological 3D Printing, Hangzhou 310018, China

**Keywords:** high-fidelity printing, GCode information, fidelity assessment, target model, defect characterization map

## Abstract

To address the challenges associated with achieving high-fidelity printing of complex 3D bionic models, this paper proposes a method for spatially resolved defect characterization and fidelity assessment. This approach is based on 3D printer-associated optical coherence tomography (3D P-OCT) and GCode information. This method generates a defect characterization map by comparing and analyzing the target model map from GCode information and the reconstructed model map from 3D P-OCT. The defect characterization map enables the detection of defects such as material accumulation, filament breakage and under-extrusion within the print path, as well as stringing outside the print path. The defect characterization map is also used for defect visualization, fidelity assessment and filament breakage repair during secondary printing. Finally, the proposed method is validated on different bionic models, printing paths and materials. The fidelity of the multilayer HAP scaffold with gradient spacing increased from 0.8398 to 0.9048 after the repair of filament breakage defects. At the same time, the over-extrusion defects on the nostril and along the high-curvature contours of the nose model were effectively detected. In addition, the finite element analysis results verified that the 60-degree filling model is superior to the 90-degree filling model in terms of mechanical strength, which is consistent with the defect detection results. The results confirm that the proposed method based on 3D P-OCT and GCode can achieve spatially resolved defect characterization and fidelity assessment in situ, facilitating defect visualization and filament breakage repair. Ultimately, this enables high-fidelity printing, encompassing both shape and function.

## 1. Introduction

Three-dimensional (3D) printing, as a pivotal additive manufacturing technology, has extensive applications across the medical, aerospace and education fields [1]. Despite its broad potential, particularly in complex 3D bioprinting, challenges persist, encompassing printing defects and low structural fidelity. These issues significantly impact the quality and stability of printed constructs [2,3]. Low structural fidelity not only impedes the complete restoration of details and accuracy in the target model but also affects overall performance [4,5]. Common printing defects, including material unevenness, poor interlayer bonding, cracks, surface roughness and deformation, pose limitations to the widespread adoption and advancement of 3D printing [6,7,8]. It is of vital importance to ensure precise material deposition in order to guarantee the structural integrity and functionality of complex hierarchical structures in natural tissues during their complete construction. For example, geometric cues in tissue engineering constructions can affect hepatocyte maturation [9], stem cell differentiation [10,11,12] and muscle tissue regeneration [13]. Deposition defects, such as excessive filling of pores and inconsistent pore geometries, can physically impede the process of bone ingrowth into bone scaffolds [14]. Moreover, achieving high-fidelity 3D bioprinting is vital for its widespread application in the clinical field. 3D bioprinting is not only useful for in vivo implantation [15,16,17,18], but also for in vitro tissue models used in drug screening and disease modelling [19].

The challenges of achieving high-fidelity 3D printing present obstacles to the printing and preparation of intricate biological structures. In this context, in situ monitoring emerges as a crucial tool for defect detection and quality assessment [20]. In situ monitoring and defect detection of the printed structure enables the adjustment of printing parameters, such as temperature, pressure and speed, in order to minimize defects and improve structural fidelity [21]. Furthermore, in situ monitoring and defect detection enables users to promptly identify issues during the printing process, facilitating timely interventions to prevent print failures and material wastage [22]. Consequently, in situ monitoring techniques for accurate defect detection and rapid visualization of 3D printing defects are expected to facilitate the extensive application and development of 3D printing technology across various fields, particularly in the realm of 3D bioprinting.

In Fused Deposition Modeling (FDM), optical sensors have been widely used for process monitoring and defect detection, such as of short fiber Bragg grating (FBG) [23], with cameras [24] and infrared thermography [25]. Laser displacement scanners are capable of acquiring high-precision point cloud data, although this is limited to surface point information and is susceptible to environmental light and reflections. Therefore, controlled surroundings are necessary for the acquisition of accurate data [26,27,28]. Camera-based monitoring methods, such as digital image correlation [29] and structured light microscopy [30], exhibit restricted depth perception, potentially missing certain 3D details, and are affected by lighting conditions, resulting in diminished material–background contrast [31,32,33]. Optical coherence tomography (OCT) is a non-destructive, label-free, high-resolution and fast tomographic imaging technique that has been widely applied in biomedical and industrial inspection fields [34,35]. For instance, OCT has been integrated into a selective laser sintering (SLS) system to detect sub-surface defects, thereby identifying failures in the construction process [36]. DePond PJ, et al. employed OCT to quantify surface roughness during the laser powder bed fusion (L-PBF) process, integrating layer height measurements and defect detection, including the identification of large splatters [34]. It is regrettable that the current defect detection methods with OCT have limitations in universality, and it is difficult to adapt them to different 3D printing technologies and materials, including SLS, PBF, droplet-based, extrusion-based and photocuring-based printing. This study proposes a defect detection method, through a comparison and analysis of a target model and a reconstructed model, which is universal and independent of the specific additive manufacturing implementation method. In order to provide a comprehensive explanation of the method, this study has chosen to provide a detailed explanation of extrusion-based 3D bioprinting.

In our previous studies, we proposed a 3D extrusion-based bioprinter integrated with OCT (3D P-OCT). 3D P-OCT enables the acquisition of large field-of-view full-depth images and multi-parameter characterization, meeting the imaging requirements for large-scale structures [21]. However, spatially resolved defect characterization and visualization during the printing process are of paramount importance for evaluating print structure fidelity and adjusting print parameters. It can even provide suggestions for terminating the printing process in advance, thereby avoiding material waste and print failure [37,38]. Defect detection techniques based on target models and actual printed results are compatible with complex and irregular print models, reproducible and expected to meet the reliability and complexity requirements of clinical and disease model construction [33].

Building on previous work [21], this study presents a spatially resolved defect characterization map for any irregular print path using extrusion-based 3D bioprinting combined with OCT. First, a target model generation mechanism based on GCode information is proposed to automatically generate target model maps. Then, OCT-reconstructed model maps (representing actual print results) are generated and compared with the target model maps. This enables spatially resolved visualization of defects and fidelity analysis using the structural similarity index. In addition, secondary print information is automatically generated for defect repair, covering issues such as filament breakage and under-extrusion. This includes print coordinates and parameters such as print speed and air pressure based on a “pre-built feedback mechanism” [22]. The proposed method is validated using different models, print paths and materials to assess print fidelity and provide feedback information.

## 2. Materials and Methods

### 2.1. System

This study employed a self-developed 3D P-OCT system (Silicone, Hydroxyapatite (HAP), Polycaprolactone (PCL), and Regenovo Bio-Architect PX, Hangzhou Regenovo Biotechnology Co., Ltd., Hangzhou, China). This system integrates a 3D bioprinter with OCT and its performance has been validated in previous studies [21]. Briefly, 3D P-OCT incorporates a self-developed swept-source OCT (SS-OCT) module. The OCT probe is mounted next to the print extrusion nozzle for in situ process monitoring. Through a two-dimensional high-speed galvanometer scanning module, a single 3D P-OCT dataset can cover an area of 10 mm (x) × 10 mm (y) × 6.28 mm (z).

### 2.2. Printing Materials and Printing Path

In this study, three materials with wide applications and significant importance in the fields of bioengineering and medicine were selected, including hydroxyapatite (HAP), silicone and polycaprolactone (PCL). HAP stands out as the primary mineral building block of human bones and teeth and has immense utility, especially in bioprinting, bone repair and the production of biodegradable scaffolds [39]. Silicone is known for its outstanding physicochemical properties and robust environmental adaptability, enabling it to function effectively under complex conditions. The biodegradability of PCL is of great importance for bioprinting, tissue engineering and drug delivery systems. Its gradual post-function degradation minimizes its impact on the body’s internal environment, making it a key element in biomedical advances.

A series of print paths were designed using Computer Aided Design (CAD), including simple single-layer and multi-layer gradient spacing models, as well as complex nose and ear models. For the single- and multi-layer gradient spacing models, HAP was selected as the print material using a nozzle with an internal diameter of 0.41 mm. The print pressure was set at 0.18 MPa and the target layer thickness was 0.25 mm. For the straight path, the print speed was set to 10 mm/s, while the print speed around the turnarounds was set to 12 mm/s to reduce material accumulation. The size of the single-layer gradient model was 9 mm × 9 mm × 0.25 mm and the multi-layer gradient model was 9 mm × 9 mm × 5 mm. For the nose model, silicone was selected as the material and a needle with an internal diameter of 0.21 mm was used. The print pressure was set at 0.15 MPa and the printing speed was 10 mm/s. The size of the model was 10 mm × 18 mm × 6 mm, with a layer thickness of 0.18 mm. For the ear model, PCL was selected as the print material and a needle with an internal diameter of 0.15 mm was used. The print pressure was set at 0.55 MPa and the print speed was 2 mm/s, with temperature control at 130 °C. The size of the model was 11.04 mm × 16.53 mm × 5.42 mm, with a layer thickness of 0.18 mm. In order to investigate the relationship between filling path and structural accuracy, two different filling strategies were used in this experiment: 60-degree and 90-degree filling. At the same time, key print parameters were optimized, including print pressure and speed and temperature control of the print material. The aim of this approach was not only to improve structural fidelity, but also to ensure the mechanical performance of the printed structures.

### 2.3. Generating Target Models Based on Printing Information

**Figure 1:**
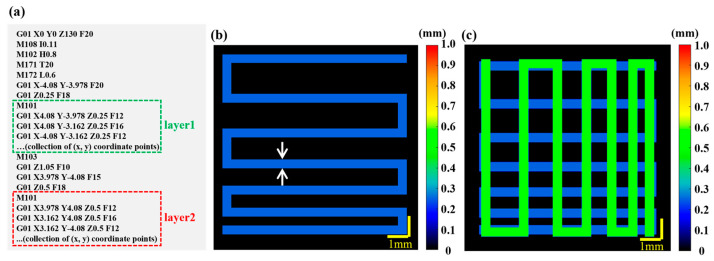
Generation of the target model based on GCode information. (**a**) GCode script. The green and red dashed boxes represent the X–Y plane coordinate sequences of the first and second layers, respectively. (**b**) Target model of Layer 1 with the target height (0.25 mm) and the target width (0.41 mm) information. Dual white arrows indicate width. (**c**) Target model of layer 1 (the deep blue channel) and layer 2 (the green channel). The target height is 0.5 mm and the target width is 0.41 mm for Layer.

The relationship between print parameters and the target layer thickness and filament diameter is calibrated by the pre-built feedback mechanism for printing guidance [22]. The pre-experiment results are provided in the Supplementary Materials (Figure S1). GCode instructions include printing parameters such as print speed, print pressure, print nozzle spatial coordinates, filament advance and filament retraction, as shown in Figure 1a and Figure S2 in the Supplementary Materials. With the combination of the pre-built feedback mechanism and the GCode information, the target model can be generated. The specific steps are as follows:

Firstly, a 2D target model map (X−Y) is generated based on the field of view of the 3D P-OCT data acquisition and the GCode information. Depending on the field of view, the 2D target model’s space size in the X and Y directions is set to [−5, 5] (or [−9.5, 9.5] for wide–field imaging). According to the acquisition parameters, the 2D target model space is then interpolated at equal intervals of 1024 × 1024. Secondly, at the same Z coordinate $Z_{k}\;$(k = 1, 2, 3, ...), the sequence of X–Y plane coordinates ($X_{i}$, $Y_{i}$) (i = 1, 2, 3, ...) is extracted from the GCode instructions. These coordinates are then plotted sequentially in the aforementioned 2D target model space. The Bresenham algorithm [40] is used to interpolate the points between ($X_{i}$, $Y_{i}$) and ($X_{i+1}$, $Y_{i+1}$) in the 2D target model space and the 2D target path can be obtained with all points after interpolation. Then, based on the print information between ($X_{i}$, $Y_{i}$) and ($X_{i+1}$, $Y_{i+1}$) in the GCode instructions (including print speed and pressure), the target print parameters for that segment (including filament height and width) can be obtained. This allows a 2D target model map to be generated at the current Z coordinate, as shown in Figure 1b. Finally, to obtain target path/model images at multiple Z coordinates $Z_{k}$ (k = 1, 2, 3...), it is only necessary to combine the aforementioned multiple 2D target model maps using Z-related color coding, as shown in Figure 1c.

### 2.4. Reconstruction of Printed Models Based on 3D P-OCT

Although the calibration results from the pre-print experiment help to improve the fidelity of the printed structure, in situ monitoring methods are still needed during the 3D printing process for defect detection and quantitative assessment. In this study, a “print-imaging” alternating mode was adopted for process monitoring of 3D printed constructs. In each cycle, after the end of printing, each defect is detected and fixed. It should be emphasized that coordinate calibration between the printing system, the imaging system and the imaging space is performed to ensure accurate printing, defect detection and feedback. Firstly, 3D P-OCT is utilized to acquire data of the base layer and record the device coordinates during acquisition. Then, based on the 3D P-OCT data, the image coordinates of the base layer are extracted. During printing, based on the penetration depth of the printing material for 3D P-OCT, data are collected after each layer or several layers are printed and the device coordinates are recorded for each acquisition. The X–Y coordinates are the same as the base layer data acquisition coordinates, while the Z coordinate is adjusted based on the thickness of the newly printed scaffold since the last acquisition. Finally, surface point detection is performed on 3D P-OCT datasets and the positions of the surface points at each X–Y coordinate are recorded. This is further combined with the device coordinates corresponding to the acquisition to generate the thickness distribution of each round of the newly printed structure, which is used to reconstruct the printed model image. The thickness distribution of the reconstructed printing model can be calculated using the following formula:

(1) $${Thickness}_{Print(i)}=\left(Z_{System\_Base}+Z_{Set\_Height(i)}-Z_{Imaging\_Print(i)}\right)\times ∆Z$$

Here, $Z_{System\_Base}$ is the surface point Z coordinates of the printing platform in the 3D P-OCT data. $Z_{Set\_Height(i)}$ represents the number of pixels by which the print platform moves downward along the Z-axis during the i−th round of printing. $Z_{Imaging\_Print(i)}$ is the surface point matrix of the current layer material in the 3D P-OCT data and ∆Z is the pixel resolution in the Z-direction in the 3D P-OCT data. In addition, this processing method can also eliminate errors in judging the height of the printed structure caused by an uneven base.

**Figure 2:**
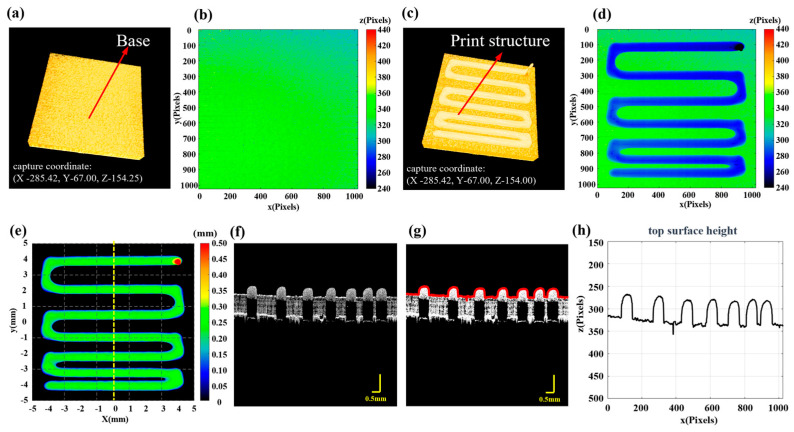
Reconstruction of the printed model. (**a**) 3D P-OCT results of the print platform, with the system coordinates for data acquisition. (**b**) The Z coordinates of surface points for the base layer. (**c**) 3D P-OCT results of the first layer with the system coordinates for data acquisition; the difference in Z values represents the height setting of the current print layer. (**d**) The Z coordinates of surface points for the first layer. (**e**) The reconstructed printed model map. (**f**) Two-dimensional cross-sectional image corresponding to the yellow dashed line in (**e**). (**g**) Red surface points extracted from (**f**). (**h**) Height profile corresponding to the red surface points in (**g**).

Figure 2 further demonstrates the process of using the formula. Figure 2a,c show the 3D P-OCT results for the base and the first round of printing, respectively. Figure 2b and Figure 2d are the surface point height maps of Figure 2a and Figure 2c, respectively. Figure 2e shows the reconstructed printed model with thickness calculation information. Figure 2f is a two-dimensional cross-sectional image at the dashed line position of Figure 2e, Figure 2g is the surface point extraction image of Figure 2f and Figure 2h is the height profile corresponding to Figure 2g. Figure S3 in the Supplementary Materials shows a detailed example diagram of the formulae. Based on the above method, it is possible to perform 3D P-OCT imaging of the printed structure in each round and obtain the reconstructed printed model during the printing process. This enables subsequent monitoring of print quality and defect detection in 3D printing.

### 2.5. Defect Characterization Map and Fidelity Assessment

**Figure 3:**
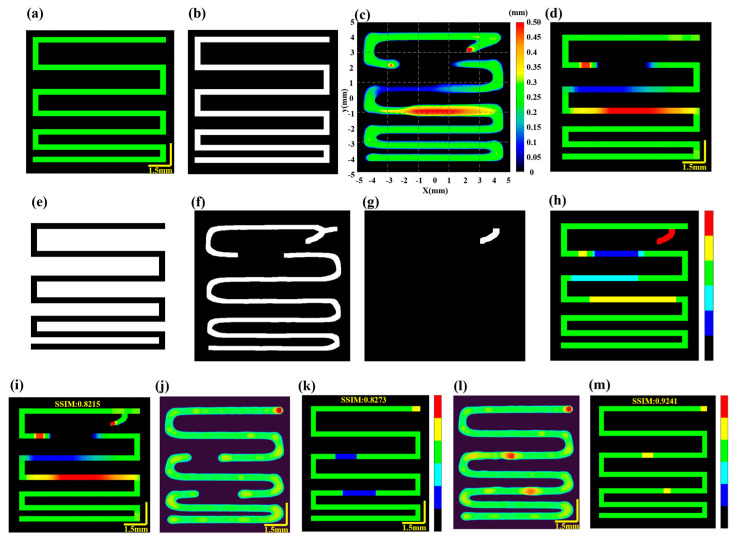
Print defect detection. (**a**) Target model. (**b**) Target model mask. (**c**) Reconstructed printed model. (**d**) Reconstructed printed model within the target path. (**e**) Inverted target model mask. (**f**) Resulting image from skeleton extraction and dilation of (**c**). (**g**) Stringing defect results. (**h**) Defect characterization map: background (black), normal extrusion (green), under-extrusion (cyan), fila-ment breakage (dark blue), over-extrusion (yellow) and stringing (red). (**i**) Final reconstructed printed model within and outside the target path. (**j**) Reconstructed printed model before filament breakage repair. (**k**) Defect characterization map before filament breakage repair. (**l**) Reconstructed printed model after filament breakage repair. (**m**) Defect characterization map following filament repair.

In the process of extrusion-based bioprinting, defects significantly affect the fidelity of the printed structure and hinder the wider application of the technology in tissue engineering and medical fields. This study mainly discusses four different defects, including material accumulation, under-extrusion, filament breakage inside the print path and stringing outside the path. Based on the comparison between the aforementioned target model maps and the reconstructed 3D printed models, this study proposes a method for detecting and identifying thickness defects in the 3D printing process under any irregular path. Firstly, a target model mask is generated (Figure 3b) based on the target model map (Figure 3a) and this mask is then point-multiplied with the reconstructed printed model (Figure 3c) to obtain the reconstructed printed model inside the target path (Figure 3d).

The operator then defines the defect status based on the target thickness and the actual thickness along the central path of the target model (Figure 3a) and the reconstructed printed model within the target path (Figure 3d), taking into account the tolerable error. For example, the definition of print status in this study is as follows:

Normal printing:

${Thickness}_{Target}-0.05\;mm\leq {Thickness}_{Print1}\leq {Thickness}_{Target}+0.05\;mm;$

Under-extrusion: $0<{Thickness}_{Print1}<{Thickness}_{Target}-0.05\;mm;$

Over-extrusion: ${Thickness}_{Print1}>{Thickness}_{Target}+0.05\;mm;$

Filament breakage: ${Thickness}_{Print1}=0\;and\;{Thickness}_{Target}\neq 0;$

Finally, the mask of the target model mask is inverted (Figure 3e) to monitor for out-of-path defects. The skeleton of the reconstructed printed model is extracted and expanded (Figure 3f). The dot product of Figure 3e,f after opening operations is displayed in Figure 3g, demonstrating material deposition outside the print path. Based on Figure 3g, stringing defects are evaluated as follows:

Stringing: ${Thickness}_{Print1}^{\prime }>0$.

Specifically, based on the alignment of the target model map and the reconstructed printed model, the states of normal extrusion, under-extrusion, over-extrusion, filament breakage and stringing are identified according to the aforementioned criteria. Different types of print defects are then displayed in the path-related defect characterization map (Figure 3h). In the defect characterization map, the background is black, normal extrusion is green, under-extrusion is cyan, filament breakage is dark blue, over-extrusion is yellow and stringing defects are red. Based on the defect characterization map, it is possible to identify the states and locations of defects. Therefore, the defect detection mechanism proposed primarily focuses on deviations between the printed structure and the design model and is not directly related to the printing parameters themselves, such as the nozzle size, printing speed and pressure. Different combinations of needle sizes and printing parameters generally correspond to different target models and thus the method exhibits good robustness. For detailed information, please refer to Figures S4 and S5 in the Supplementary Materials.

To further quantitatively evaluate the fidelity of 3D printed structure, the binarization result of the defect characterization map (Figure 3h) is dot-multiplied with the reconstructed print model (Figure 3c), generating the reconstructed print model map inside and outside the path (Figure 3i). The structural similarity index (SSIM) is then calculated between Figure 3a,i, with the moving window size determined by the target filament diameter. The average SSIM value is used to evaluate the deviation between the target model and the reconstructed 3D printed model, characterizing the fidelity of the printed structure. The expression for SSIM is:

(2) $$SSIM\left(x,y\right)=\frac{\left(2\mu_{x}\mu_{y}+c_{1}\right)\left(2\sigma_{xy}+c_{2}\right)}{\left(\mu_{x}^{2}+\mu_{y}^{2}+c_{1}\right)\left(\sigma_{x}^{2}+\sigma_{y}^{2}+c_{2}\right)}$$

Here, x and y represent the target model map and the reconstructed printed model map, respectively. $\mu_{x}$ and $\mu_{y}$ denote the mean values of x and y. $\sigma_{x}$ and $\sigma_{y}$ represent the standard deviations of x and y. $\sigma_{xy}$ is the covariance of x and y. $c_{1}$ and $c_{2}$ are constants used to enhance stability. The average SSIM serves as the fidelity value of the printed structure. This approach is particularly useful for addressing filament breakage and under-extrusion defects, as it enables the identification of defect locations, which can the be used to optimize printing results through parameter adjustments or to repair filament breakage with a secondary printing process. Figure 3j,k illustrates the reconstructed printed model with filament breakage defects and its corresponding defect characterization map. Figure 3l,m display the reconstructed printed model map and the defect characterization map after the filament breakage defects have been repaired through secondary printing. For instance, the fidelity of the reconstructed printed model displayed in Figure 3j is 0.8273, while the fidelity after repair is 0.9241 (Figure 3k), representing an improvement of 11.70%.

### 2.6. Statistical Analysis

In this study, data processing and analysis were conducted using MATLAB 2020a software and the 3D perfusion map was rendered using Amira (ZIB, Indeed-Visual Cocepts GmbH, Berlin, Germany). All results are expressed as the mean ± standard error of the mean.

## 3. Results

### 3.1. Multilayer Gradient Spacing HAP Scaffold

**Figure 4:**
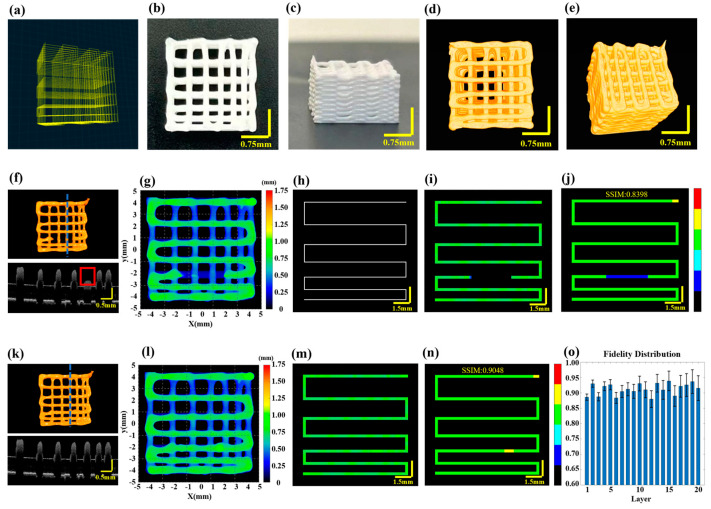
Multilayer structure with gradient spacing before and after filament breakage repair feed-back. (**a**) The designed printing path for a ten-layer gradient spacing pattern. (**b**) Side view of the printed scaffold. (**c**) Top view of the printed scaffold. (**d**,**e**) Optical coherence tomography (3D P-OCT) reconstruction results of the scaffold. (**f**) Top: 3D P-OCT reconstruction results of the first three layers of the scaffold with filament breakage defects; Bottom: Cross-sectional image at the blue dashed line position of the third layer. (**g**) Height map of the reconstructed printed model. (**h**) Printing path diagram of the target layer. (**i**) Height map of the reconstructed printed model within the target path before filament breakage repair. (**j**) Defect characterization map of the third layer prior to filament breakage repair: background (black), normal extrusion (green), under-extrusion (cyan), filament breakage (dark blue), over-extrusion (yellow) and stringing (red). (**k**) Top: 3D P-OCT data of the first three layers of the scaffold following filament breakage repair; Bottom: Cross-sectional image at the blue dashed line position of the third layer. (**l**) A height map of the reconstructed printed model after filament breakage repair. (**m**) A reconstructed printed model within the target path after filament breakage repair. (**n**) A defect characterization map of the third layer after filament breakage repair: background (black), normal extrusion (green), under-extrusion (cyan), filament breakage (dark blue), over-extrusion (yellow) and stringing (red). (**o**) The structural fidelity of the different layers.

To validate the effectiveness of the proposed method for defect characterization and fidelity assessment in multilayer printing, HAP material was utilized to print a 20-layer scaffold with gradient spacing. The printer nozzle had an inner diameter of 0.41 mm, the printing pressure was set to 0.18 MPa, the printing speed for straight paths was set at 10 mm/s and at corners, it was increased to 12 mm/s to reduce material accumulation. The target layer thickness was 0.25 mm and the model dimensions were set to 9 mm × 9 mm × 5 mm. The printing path is illustrated in Figure 4a and detailed printing parameters can be found in Section 2.2. Figure 4b,c display the physical images of the scaffold after optimization through defect detection and feedback control.

Given that the effective penetration depth of 3D P-OCT in HAP is approximately 0.35 mm, an alternating “print-imaging” mode was employed. Subsequently, 3D data were longitudinally stitched together to achieve wide-field 3D P-OCT reconstruction results, as illustrated in Figure 4d,e. The overall size of the scaffold was 8.873 mm × 8.895 mm × 5.132 mm. Unfortunately, filament breakage occurred during the printing of the third layer due to abnormal air pressure. The 3D P-OCT reconstruction results of the first three layers are presented at the top of Figure 4f. The blue dashed line corresponds to the cross-sectional image at the bottom of Figure 4f, with the red rectangle indicating filament breakage defects. Figure 4g shows the corresponding reconstructed printed model. By combining the target path in Figure 4h, printing parameters and the reconstructed printed model within the target path in Figure 4i, we obtained the characterization map in Figure 4j. The filament breakage measured 3.78 mm in length, with a structural fidelity of 0.8398.

Based on the aforementioned information, feedback was provided regarding the repair of filament breakage. A secondary printing was conducted from the position (−1.75, −2.142, 0.75) to (1.85, −2.142, 0.75) in order to address the filament breakage defect. The results following the repair through secondary printing are displayed in Figure 4k–o, including Figure 4k for the 3D P-OCT reconstruction results, Figure 4l for the reconstructed printed model, Figure 4m for the reconstructed printed model within the target path and Figure 4n for the defect characterization map. Following the repair, the structural fidelity increased to 0.9048. Upon completion of the repair of the filament breakage in the third layer, the remaining layers were printed. Figure 4o illustrates the structural fidelity of the different layers, with an average structural fidelity of 0.91 ± 0.02.

### 3.2. Large-Scale Silicone Scaffold for the Nose

**Figure 5:**
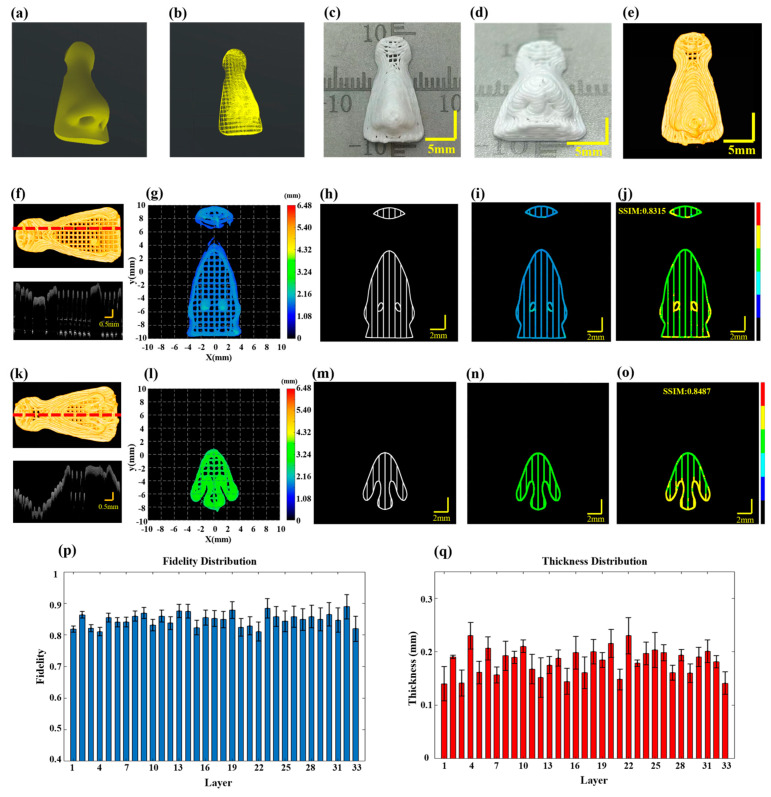
Defect detection in multilayer nose structure. (**a**) The designed nose model. (**b**) The prin-ing path for the nose. (**c**,**d**) Actual images of the printed nose. (**e**) 3D P-OCT image data of the nose. (**f**) Top: 3D P-OCT reconstruction image of the 10-layer scaffold; Bottom: A cross-sectional image corresponding to the red dashed line position of the 10th layer. (**g**) Height map of the reconstructed printed model of layers 9–10 of the nose. (**h**) An image of the print path of the 10th layer. (**i**) An image of the reconstructed printed model within the target path of the 10th layer. (**j**) Defect characterization map of the 10th layer: background (black), normal extrusion (green), under-extrusion (cyan), filament breakage (dark blue), over-extrusion (yellow) and stringing (red). (**k**) Top: 3D P-OCT data of the 18-layer scaffold; Bottom: Cross-sectional image corresponding to the red dashed line position of the 18th layer. (**l**) Height map of the reconstructed printed model of layers 17–18 of the nose. (**m**) Printing path image of the 18th layer. (**n**) Reconstructed printed model within the target path of the 18th layer. (**o**) Defect detection result map of the 18th layer: background (black), normal extrusion (green), under-extrusion (cyan), filament breakage (dark blue), over-extrusion (yellow) and stringing (red). (**p**) Statistical values of structural fidelity for each layer. (**q**) Statistical values of filament thickness for each layer.

When printing intricate biomimetic models with irregular shapes, there is an increased likelihood of material accumulation defects, particularly at turnarounds with greater curvature. To demonstrate the applicability of the proposed method for defect detection to any irregular printing path, a biomimetic nose model was printed using silicone material with a nozzle inner diameter of 0.21 mm, printing pressure set at 0.15 MPa with a printing speed of 10 mm/s and each layer thickness set at 0.18 mm. The overall design dimensions of the model were 10 mm × 18 mm × 6 mm, as shown in Figure 5a, following the printing path shown in Figure 5b. The completed biomimetic nose structure is presented in Figure 5c,d, with an overall size of 10.424 mm × 18.384 mm × 5.868 mm. The wide-field imaging mode of 3D P-OCT was also activated. The final 3D P-OCT reconstruction of the printed nose structure following horizontal and vertical stitching is presented in Figure 5e. Once the 10th layer of printing was completed, 3D P-OCT data were acquired. The reconstructed image is shown at the top of Figure 5f, with the corresponding cross-sectional image at the bottom of Figure 5f. The reconstructed printed model is shown in Figure 5g. Figure 5h displays the target path, with the reconstructed printed model within the target path shown in Figure 5i. Subsequently, a defect characterization map was generated, displayed in Figure 5j. The results indicated the presence of over-extrusion defects at the nostril and contour areas with larger curvature, featuring significantly higher average heights of 2.12 mm and 2.08 mm, respectively. Additionally, the structural fidelity of the 10th layer was 0.8315. Upon completion of the 18th layer of printing, the 3D P-OCT dataset was acquired. The reconstructed image is presented at the top of Figure 5k and the corresponding cross-sectional image is found at the bottom of Figure 5k. Figure 5l depicts the corresponding reconstructed printed model. The defect characterization map (Figure 5o) was generated by combining the target path in Figure 5m with the print parameters and the reconstructed printed model within the target path in Figure 5n. The results revealed that over-extrusion defects persisted at the nostrils with the larger curvature. The structural fidelity of the 18th layer was measured at 0.8487. Figure 5p provides the average layer thickness of each layer, while Figure 5q illustrates the structural fidelity of each layer. The results indicated that the closer the average layer thickness was to the designed value of 0.18 mm, the higher the structural fidelity, particularly for the 32nd layer. The overall structural fidelity was 0.85 ± 0.02. In the Supplementary Materials, Figure S6 presents the 3D reconstruction rendering image of the nose model after horizontal and vertical stitching.

### 3.3. Defect Detection and Mechanical Comparison of the Ear Model

**Figure 6:**
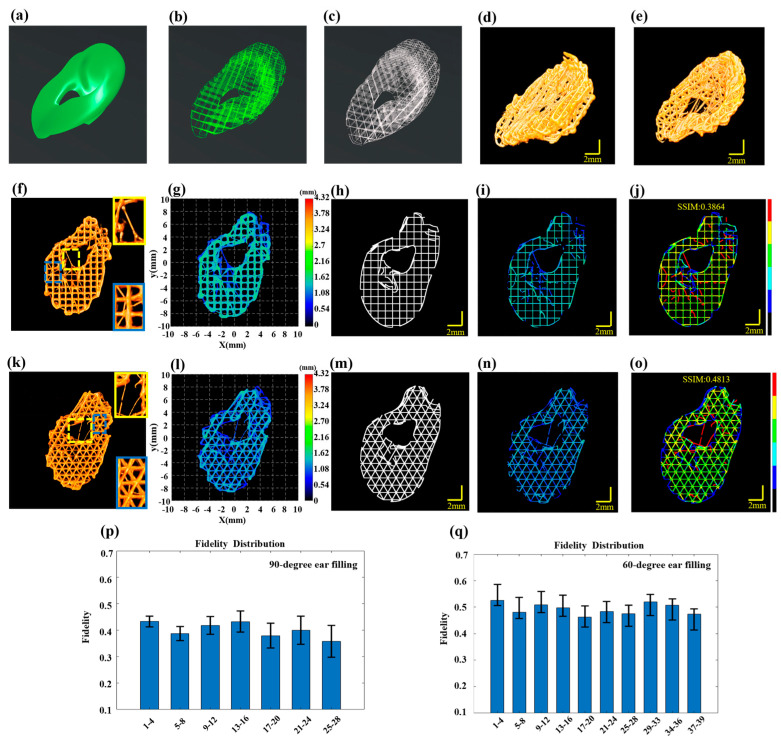
Multilayer structure defect detection in the ear. (**a**) The designed ear model. (**b**) The 90-dgree fill printing path for the ear. (**c**) The 60-degree filling printing path for the ear. (**d**) 3D P-OCT reconstructed printing result for the 90-degree filling. (**e**) 3D P-OCT reconstructed printing result for the 60-degree filling. (**f**) 3D P-OCT reconstruction results for layers 5–8 in the 90-degree filling, with enlarged views in yellow and blue boxes highlighting the stringing defects. (**g**) Reconstructed printed model of layers 5–8 in the 90-degree filling. (**h**) Printing path of layers 5–8 in the 90-degree filling. (**i**) Reconstructed printed model within the target path of layers 5–8 in the 90-dgree filling. (**j**) Defect characterization map of 90-degree filling: background (black), normal extrusion (green), under-extrusion (cyan), filament breakage (dark blue), over-extrusion (yellow) and stringing (red). (**k**) 3D P-OCT reconstruction results for layers 5–8 in the 60-degree filling with enlarged views in yellow and blue boxes highlighting the stringing defects. (**l**) Reconstructed printed model of layers 5–8 in the 60-degree filling. (**m**) Printing path of layers 5–8 in the 60-dgree filling. (**n**) Reconstructed printed model within the target path of layers 5–8 in the 60-degree filling. (**o**) Defect characterization map of 60-degree filling: background (black), normal extrusion (green), under-extrusion (cyan), filament breakage (dark blue), over-extrusion (yellow) and stringing (red). (**p**) Structural fidelity assessment results for every four layers in the 60-degree filling. (**q**) Structural fidelity assessment results for every four layers in the 90-degree filling.

In order to verify the robustness of the proposed method for defect detection across various printing materials, models and paths, we employed PCL to print an ear model (Figure 6a). Due to the high viscosity of PCL and its susceptibility to stringing defects during high-temperature printing, particularly outside the designated printing paths, a needle with a smaller inner diameter of 0.15 mm was employed to achieve high-precision printing. The printing pressure was set at 0.55 MPa, with a printing speed of 2 mm/s and the temperature controlled at 130 °C. The layer thickness was set at 0.18 mm. The overall design dimensions of the model were 11.04 mm × 16.53 mm × 5.42 mm. To study the relationship between the fill path and structural accuracy, this experiment used 60-degree and 90-degree fills for comparative analysis. The printing paths for the ear model with 60-degree and 90-degree infill are shown in Figure 6b and Figure 6c, respectively. In the alternating ‘print-imaging’ mode, the large wide-field imaging mode of 3D P-OCT was also activated. The overall reconstructed images following horizontal and vertical stitching are presented in Figure 6d,e. Figure 6d showcases the result of 90-degree filling, while Figure 6e displays the result of 60-degree filling.

With 90-degree filling, 3D P-OCT data were collected after every four layers of printing. The 3D P-OCT reconstruction results of layers 5–8 are depicted in Figure 6f. The locations marked by the yellow and blue rectangular boxes indicate the presence of stringing defects. The corresponding reconstructed printed model is shown in Figure 6g. The defect characterization map (Figure 6j) was generated by combining the target path in Figure 6h with the reconstructed printed model within the target path in Figure 6i. The results demonstrate that when utilizing PCL material and a 90-degree fill path for printing, a multitude of stringing defects (red), filament breakage defects (blue) and accumulation defects (yellow) were evident, resulting in a structural fidelity of 0.3864. Structural fidelity was evaluated at four-layer intervals, as illustrated in Figure 6p, with an average structural fidelity of 0.40 ± 0.03.

Figure 6k illustrates the 3D POCT reconstruction results for layers 5–8, which reveal the presence of stringing defects at the locations marked by the yellow and blue rectangular boxes. Figure 6l depicts the corresponding reconstructed printed model. By combining the target path in Figure 6m with the reconstructed printed model within the target path in Figure 6n, the defect characterization map was generated and shown in Figure 6o. The results indicate that when using PCL material and a 60-degree filling path for printing, there were stringing defects (red), filament breakage defects (blue) and accumulation defects (yellow), resulting in a structural fidelity of 0.4813. The structural fidelity was evaluated at four-layer intervals, as illustrated in Figure 6q, with an average structure fidelity of 0.49 ± 0.02. This represents a 9% improvement compared to the 90-degree filling. In the Supplementary Materials, Figures S7 and S8 present the 3D reconstruction rendering images of the ear models with 90-degree and 60-degree filling, respectively.

**Figure 7:**
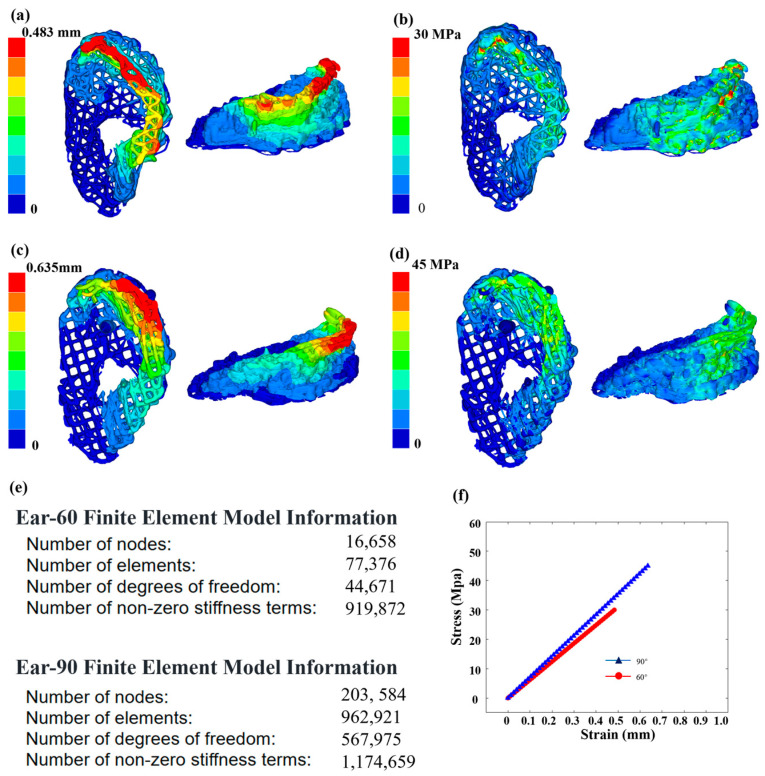
Mechanical properties of ear scaffolds. (**a**) Finite element analysis results of strain for ear model under the 60-degree filling from different perspectives. (**b**) Finite element analysis results of stress for the 60-degree filling from different perspectives. (**c**) Finite element analysis results of strain for the 90-degree filling from different perspectives. (**d**) Finite element analysis results of stress for the 90-degree filling from different perspectives. (**e**) Finite element model information for the 60-degree and 90-degree filling models. (**f**) Stress–strain curves for the ear models with 60-degree and 90-degree filling.

As shown in Figure 7, finite element analysis (FEA) was used to evaluate the impact of different fillers (60 degrees and 90 degrees) on the mechanical properties of PCL used in the ear brackets. Using Hyper Mesh 2020 software, the ear data images reconstructed by OCT technology were input and the physical properties are were according to the characteristics of the PCL material. The Young’s modulus was set to 450 MPa, the Poisson’s ratio set to 0.39 and the density set to 1.145 g/cm^3^. A tetrahedral mesh was used for the structural simulation. Considering scenarios involving the use of headphones and noise-canceling earmuffs, both the headband of the headphones and the earmuffs exert vertical pressure on the ears. To accurately assess the performance of the ear support structure in real environments, vertical pressure was selected, making the research results broadly applicable. To simulate the actual fixed state of the scaffold, fixed constraints were applied to the bottom of the model and a uniform vertical downward displacement of 0.5 mm was applied at the top. The analysis results showed that the maximum deformation displacement of the ear support with the 60-degree filler was 0.292 mm, which was significantly lower than the 0.47 mm of the 90-degree filler support. Additionally, the maximum stress of the 60-degree filler support was 9.65 MPa, lower than the 14.26 MPa of the 90-degree support. These results indicate that the ear support with the 60-degree filler performs better in terms of deformation control and stress tolerance and are consistent with the fidelity results calculated in this study.

## 4. Discussion

This study introduces a method for spatially resolved defect characterization and fidelity assessment for 3D bioprinting which compares a target model map and reconstructed model map. In situ monitoring through 3D P-OCT was employed to generate a printed structure model map, which was then compared with the target model map created using GCode and calibration information from the pre-experiments. The result was a defect characterization map that provided intuitive and spatially resolved defect information for each iteration of “print-imaging”. Notably, this approach demonstrates adaptability to complex bionic 3D bioprinting, extending beyond fixed fills or regular periodic paths. Furthermore, this method is effective in identifying various printing defects, including over- and under-extrusion, as well as filament breakage within the paths and stringing defects outside the paths. Additionally, the method has the potential to detect more concealed internal defects, such as air bubbles, in future work. In recent research, Zhang et al. developed an advanced 3D bioprinting anomaly detection system that utilizes convolutional neural networks to identify and classify defects in biological functional structures [31]. This system significantly enhances bioprinting accuracy through real-time adjustments during the printing process. However, this approach still faces challenges in utilizing 3D information and adapting to irregular complex paths. Additionally, in the previous research, 3D P-OCT was utilized to achieve high-speed, large-field and full-depth imaging, as well as real-time multiparameter quantification, thereby enhancing the structural and functional performance of bioprinted structures [21]. However, it still lacks in defect visualization and making timely decisions based on process assessment to avoid material wastage. Compared to these methods, the approach proposed in this study not only provides more comprehensive defect identification but also guides the optimization of the printing process directly through intuitive defect characterization maps, demonstrating significant practicality and broad application prospects.

The occurrence of printing defects in 3D bioprinting follows specific patterns rather than being random. Defects are more likely to manifest in certain areas, notably at the beginning and end points of the printing path, corners and turnarounds with larger curvature and regions with dense G-code nodes. Previous studies have delved into defects at the start and end points and turnarounds of lattice scaffolds, proposing corresponding repair and optimization strategies [22]. However, it is necessary to develop more accurate defect detection and visualization for arbitrary irregular printing paths, where there are more dense GCode nodes causing over-extrusion defects. Proactive measures must be taken to address over-extrusion defects, such as reducing the extrusion amount at turnarounds and adjusting the printing speed beforehand to modify acceleration. When dealing with rigid and viscous materials, which possess distinct physical properties, specific parameter settings and adjustments become essential. Consequently, the capacity to adapt parameters such as pressure and speed is advantageous in reducing the incidence of over-extrusion defects.

To address under-extrusion defects and filament breakage, the proposed method enables partial repair through secondary printing, guided by the feedback information provided. However, as previously mentioned, printing defects are prone to occurring at starting and stopping points, presenting a recurring challenge during secondary printing. Therefore, optimizing the printing parameters for these critical areas becomes particularly crucial. The utilization of the defect characterization map allows for identification of defects, such as filament breakage or under-extrusion, which reach or exceed 1.5 mm in length. In such instances, secondary printing methods are employed for the purpose of defect repair, thereby enhancing the fidelity of the scaffold. This approach has been demonstrated to be effective in minimizing the impact of printing defects on the quality of the scaffold, ensuring consistency and maintaining the accuracy of the printed scaffold.

In 3D bioprinting, the scaffold structure plays a pivotal role in cell adhesion, proliferation, differentiation and functional expression. Consequently, non-destructive testing techniques are of significant importance in assessing and ensuring the quality of cell-loaded scaffolds. Throughout the cell printing process, the focus extends beyond maintaining cell viability to ensuring that the post-printed scaffold structure positively impacts the cell state. The utilization of non-destructive testing enables the real-time monitoring of key parameters, including scaffold morphology, surface structure and material uniformity, in situ. The prompt identification and repair of defects during the printing process are facilitated, thereby ensuring the precision and consistency of the printed scaffold. Furthermore, this technology strongly supports the optimization of printing parameters, with monitoring results guiding adjustments in printing settings to further enhance the quality and fidelity of the printed scaffold. The non-destructive testing technology proposed in this study not only allows for a comprehensive assessment of the scaffold’s impact on cell states but also provides critical support for spatiotemporal iterative printing. Additionally, the proposed method is not constrained to extrusion-based printing; with minor modifications, it can be compatible with other printing technologies, including SLS, PBF, droplet-based, extrusion-based and Digital Light Processing (DLP) 3D printing, through the comparison of a target model and reconstructed model. Figure S9 in the Supplementary Materials shows the results of defect detection for DLP 3D printing.

However, the current method still exhibits limitations in certain aspects. Specifically, while it is possible to repair broken filament defects, the option of terminating the current printing process in the face of serious defects such as over-extrusion and drawing limits the scope of its application. In addition, the current “print-imaging” alternating strategy creates interruptions in the printing process, which increases the overall printing time. To improve this situation, subsequent studies could explore the use of OCT systems with higher acquisition speeds or micro-scanning probes to reduce the time required for data acquisition. On the other hand, the time-consuming process of 3D POCT data acquisition as well as the generation of defect characterization maps also affects the timeliness of defect detection. To address this challenge, we expect to improve the responsiveness of defect detection by accelerating the speed of data acquisition and processing through technological innovations. Finally, on the basis of constructing effective datasets, the application of deep learning techniques is also expected to bring significant improvements in defect detection efficiency. Through deep learning and data analysis, we may be able to identify various defects more accurately and quickly, providing strong support for the optimization of the printing process.

## 5. Conclusions

The proposed method combines a GCode-based target model with a reconstructed printed model generated using 3D P-OCT to create a defect characterization map during the printing process. This is achieved through an alternating “printing-imaging” mode. The method leverages GCode information and pre-experiment results to facilitate defect detection in irregular paths, enabling layer-by-layer visualization of printing defects and the quantitative assessment of fidelity. The defect characterization map enables the spatial localization and visualization of defects such as over-extrusion, under-extrusion and filament breakage within the printing path and stringing outside the printing path. GCode-related feedback information can be generated for the secondary printing process, with the aim of repairing under-extrusion and filament breakage, thereby enhancing printing fidelity. Additionally, the method holds promise for detecting more concealed internal defects, such as air bubbles, in future work. By adopting higher-speed OCT equipment and GPU-based data processing acceleration strategies, the timeliness of defect detection is significantly improved. Furthermore, real-time feedback strategies based on 2D OCT monitoring are explored, which involve real-time monitoring of the printing process and immediate adjustment of printing parameters to address issues arising during the process. Looking ahead, the integration of machine learning algorithms could further refine the defect detection process, automating the generation of corrective GCode adjustments and paving the way for closed-loop, self-optimizing 3D printing systems.

In conclusion, the proposed method enables the spatially resolved defect detection and visualization of defects in complex and arbitrary irregular 3D printing in situ, based on 3D P-OCT and GCode. This approach contributes to achieving high-fidelity printing, including both shape and functionality, and can significantly enhance the quality and fidelity of printed products, greatly advancing the application and development of 3D printing technology across various industries.

## Data Availability

Data are contained within the article.

## Supplementary Materials

The following supporting information can be downloaded at: https://www.mdpi.com/article/10.3390/s24113636/s1. Figure S1: The pre-built feedback mechanism. Figure S2: Generation of the target model map. Figure S3: Example diagram of formula one analysis. Figure S4: Printing and defect detection results with different needle diameters. Figure S5: Defect detection results at different printing speeds. Figure S6: 3D P-OCT reconstruction results of the nose model during the printing process with the alternating “printing-imaging” mode. Figure S7: Ear Model with 90-Degree Filling: OCT Rendering Image after Horizontal and Vertical Stitching. Figure S8: Defect detection for digital light processing (DLP) 3D printing.
