# Supplementary material for: Spatially Resolved Defect Characterization and Fidelity Assessment for Complex and Arbitrary Irregular 3D Printing Based on 3D P-OCT and GCode
Source: Sensors (Basel). 2024 Jun 4;24(11):3636. doi: 10.3390/s24113636 (PMC11175316; doi:10.3390/s24113636)
Supplement: Supplementary file 1 [file sensors-24-03636-s001.zip › sensors-2955684-supplementary.pdf]

Supplementary Information

Spatial-resolved defect detection and visualization for complex and arbitrary irregular 3D printing based on 3D P-OCT and GCode

Bowen Fan1†, Shanshan Yang1,2†*, Ling Wang1,2*, Mingen Xu1,2

[1]Hangzhou Dianzi University, Automation College, Hangzhou, Zhejiang (China)

[2]Zhejiang Provincial Key Laboratory of Medical Information and Biological 3D Printing, Hangzhou, Zhejiang (China)

†These authors contributed equally to this work.

*Correspondence To: Shanshan Yang, yangshan@hdu.edu.cn; Ling Wang, lingw@hdu.edu.cn

The Pre-Built Feedback Mechanism Based on Pre-Experiments

In the field of bioprinting, printing parameters, especially the print speed and nozzle pressure, directly affect the layer thickness and the filament diameter thereby influencing the structure fidelity. Understanding how to achieve desired layer thickness and filament diameter through different combinations of printing parameters is crucial for ensuring structure fidelity. To this end, we determined the relationship between printing parameters and the target layer thickness and filament diameter through the pre-printing experiment for printing guidance. Taking HAP as an example, a needle with an inner diameter of 0.41 millimeter was selected, and the pre-experiment was conducted with various groups of the print speed and pressure. Specifically, the pressure range

was set between from 0.14 to 0.28 MPa with intervals of 0.02 MPa, while the speed varied between 5 and 14 mm/s with 1 mm/s intervals.

The printing paths of the pre-printing experiment are shown in **Figure S1 (A1, A2)**, with the length of 8mm for each filament. One set of the printing results is illustrated in **Figure S1 (B)**. The wide field imaging capability of 3D P-OCT allows for imaging over a large field of view in 19mm × 19mm in the X-Y direction. Based on 3D P-OCT data, information about the filament height and width can be obtained, as shown in **Figure S1(C)**. Subsequently, the filament height and width values obtained under different combinations of printing speed and air pressure were plotted and shown in **Figure S1(D)** and **Figure S1(E)**, respectively. Consequently, the required printing speed and pressure parameters can be derived for the target filament height and width as well as the acceptable range of error. For instance, the green shaded area in the figure indicates the combinations of printing speed and pressure that can be selected when the acceptable range of filament width is 0.41 ± 0.10 mm and the acceptable range of filament height is 0.25 ± 0.05 mm.

Supporting Figure

Figure S1. The pre-built feedback mechanism.

Figure S2. Generation of the target model map.

Figure S3. Example diagram of formula one analysis

Figure S4. Printing and defect detection results with different needle diameters.

Figure S5. Defect detection results at different printing speeds.

Figure S6. 3D P-OCT reconstruction results of the nose model during the printing process with the alternating “printing-imaging” mode.

Figure S7. Ear Model with 90-Degree Filling: OCT Rendering Image after Horizontal and Vertical Stitching.

Figure S8. Ear Model with 60-Degree Filling: OCT Rendering Image after Horizontal and Vertical Stitching.

Figure S9. Defect detection for digital light processing (DLP) 3D printing.

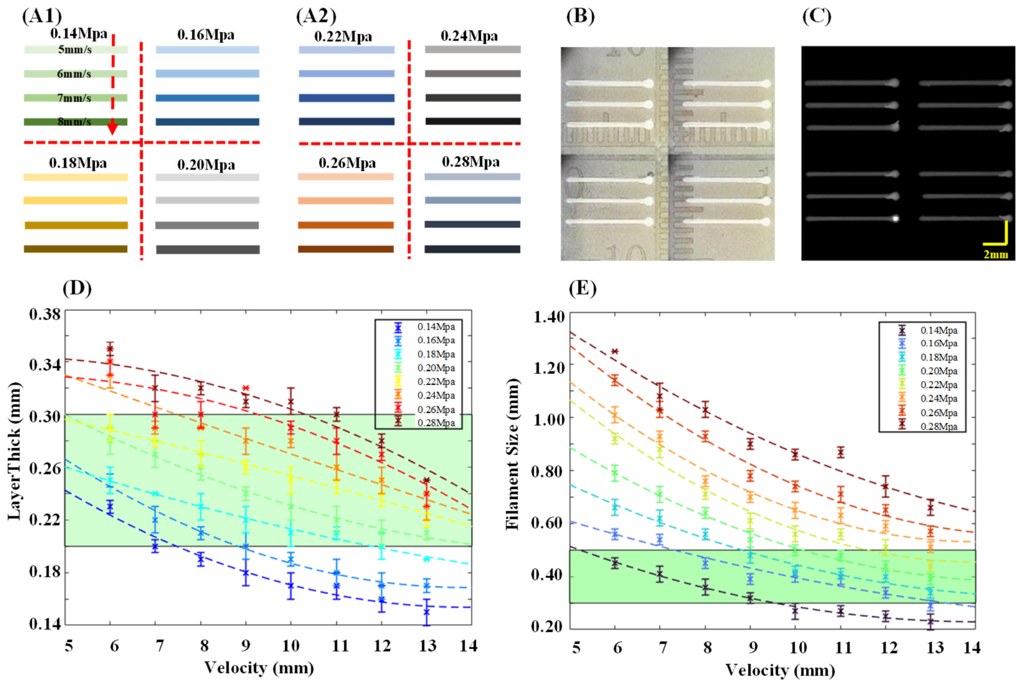

Figure S1. The pre-built feedback mechanism. (A1) and (A2) show the designed paths under different pressure and speed input parameters. (B) presents a set of print result images based on (A). (C) displays the filament height and width information results based on 3D P-OCT from (B). (D) shows the measurement results of filament diameter height under different pressures and speeds, with green representing acceptable sizes. (E) illustrates the measurement results of filament diameter width under different pressures and speeds, with green indicating acceptable sizes.

Generation of the target model map.

The 2D target model map is generated by combining the GCode information with the 3D P-OCT data acquisition strategy information.

The specific steps are shown in Figure S2:

1）Plot the XY coordinates from the GCode information within the 2D target model space, corresponding to S2(a).

2）Based on the 3D P-OCT field of view, the target area is cropped in the coordinate space. For a single field of view, the default capture interval is within the range of [-5, -5] to [5, 5]. For large field imaging, the default cropped coordinate range is [-9.5, -9.5] to [9.5, 9.5].

3）Interpolate the cropped target area based on the sampling density (number of pixels) of the 3D P-OCT. Under a single field of view, the XY pixel resolution is 9.8 micrometers with a pixel size of 1024 (x) * 1024 (y). For large field imaging, the XY pixel resolution is 19.5 micrometers with a pixel size of 1024 (x) * 1024 (y), as shown in S2(b).

4）Determine the filament diameter (width) corresponding to different speeds, pressures, and temperature parameters in the GCode information based on pre-printing suggestion experimental results. This information is used to draw the target model diagram for comparison with the 3D P-OCT reconstructed model diagram, as shown in S2(c).

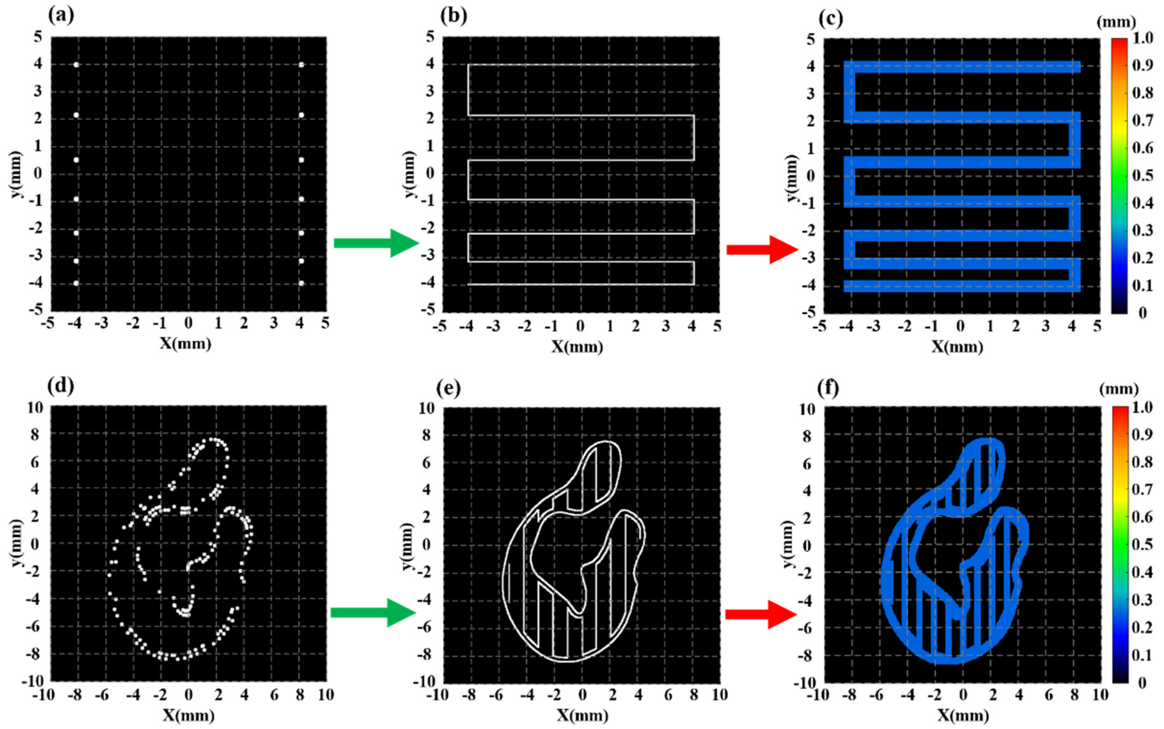

Figure S2. Generation of the target model. (a) Plot the XY coordinates of gradient spacing in the 2D target model space. (b) Plotting the print path of gradient spacing. (c) Gradient spacing target model construction results. (d) Plot the XY coordinates of the ear in the 2D target model space. (e) Plotting the print path of the ear. (f) Ear target model construction results.

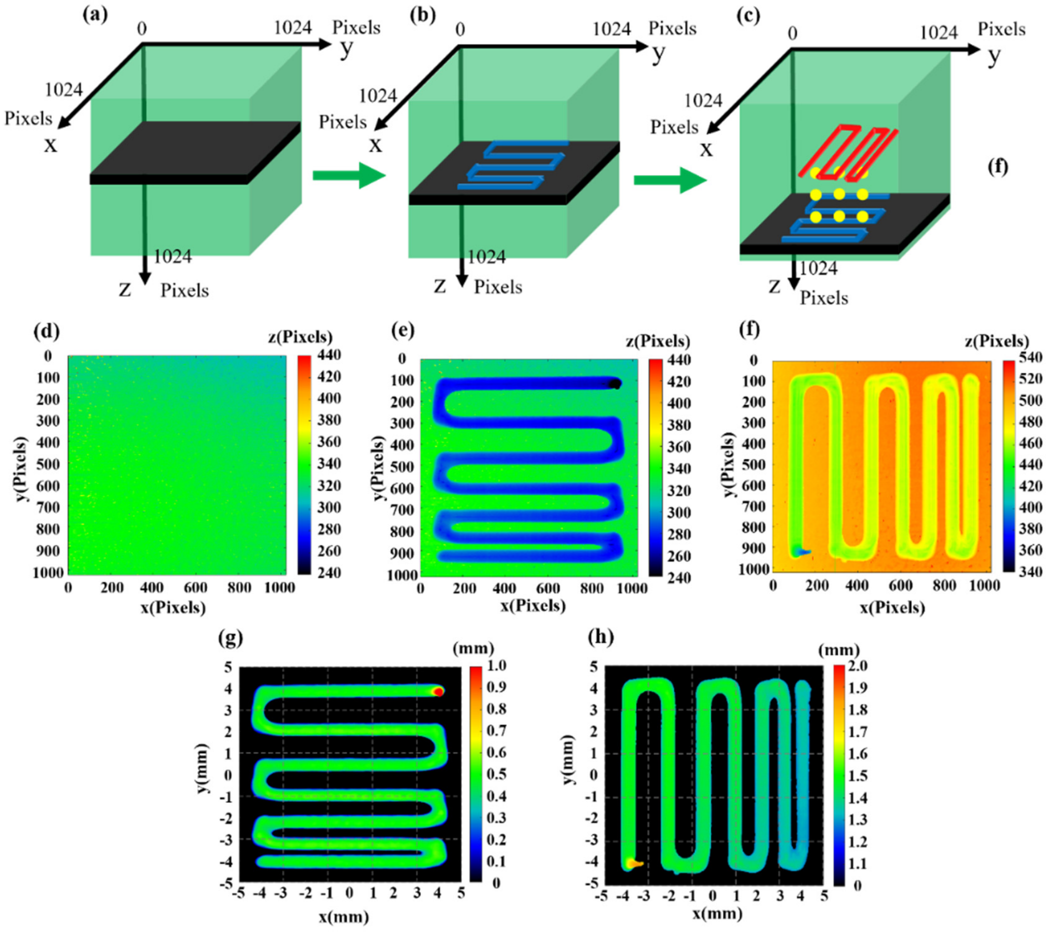

Figure S3. Reconstruction of the printed models based on 3D P-OCT. (a) 3D P-OCT data of the print platform with a data size of 1024 pixels × 1024 pixels × 1024 pixels. (b) 3D P-OCT data of the first layer of the scaffold. (c) 3D P-OCT data of the sixth layer of the scaffold. (d) 3D P-OCT surface point matrix obtained by moving the print platform down after the first layer is printed. (e) Surface point matrix of the 3D P-OCT data for the first layer of the scaffold. (f) Surface point matrix of the 3D P-OCT data for the sixth layer of the scaffold. (g) The reconstructed printed model of the first layer. (h) The reconstructed printed model of the sixth layer. The green arrow represents the downward movement of the print platform during the printing process, with the distance of movement determined by the set layer thickness.

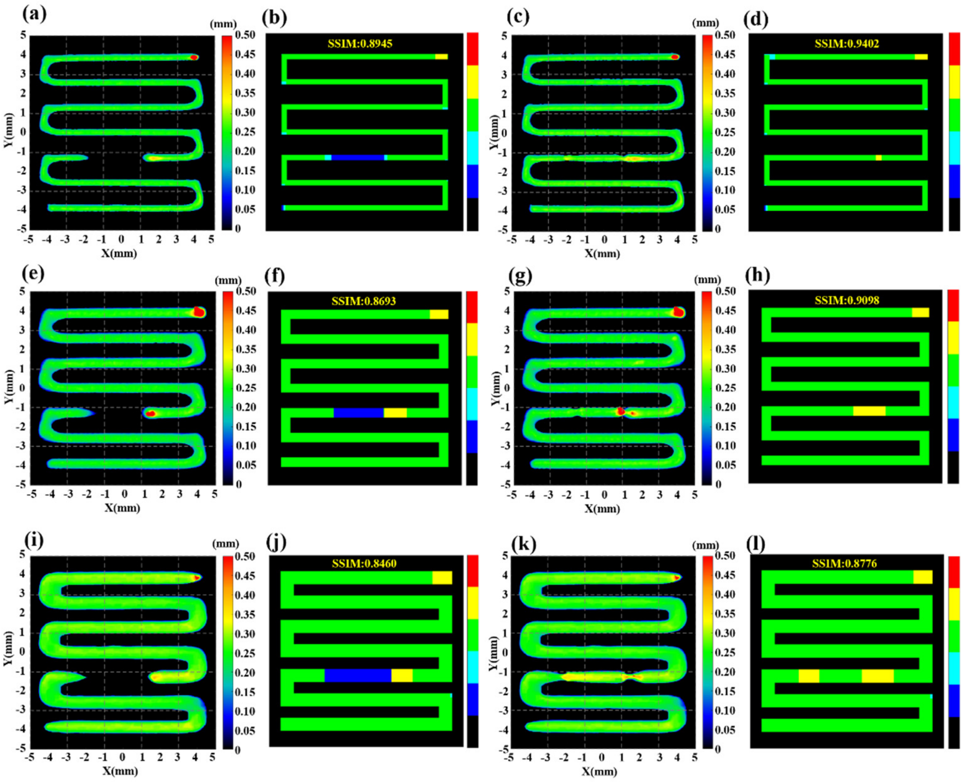

Figure S4. Printing and defect detection results with different needle diameters. Detection outcomes of printing defects using needles of various inner diameters. (a) Reconstructed printing model of the 0.26mm needle based on 3D P-OCT.(b) Defect characterization map for (a), where red indicates stringing, yellow indicates over-extrusion, green indicates normal extrusion, cyan indicates under-extrusion, dark blue indicates broken filament, and black represents the background. (c) 3D P-OCT reconstructed printing model for the broken filament repair from (a). (d) Defect characterization map for (c). (e) Reconstructed printing model of the 0.42mm needle based on 3D P-OCT.(f) Defect characterization map for (e). (g) 3D P-OCT reconstructed printing model for the broken filament repair from (e). (h) Defect characterization map for (g). (i) Reconstructed printing model of the 0.61mm needle based on 3D P-OCT.(j) Defect characterization map for (i). (k) 3D P-OCT reconstructed printing model for the broken filament repair from (i). (l) Defect characterization map for (k).

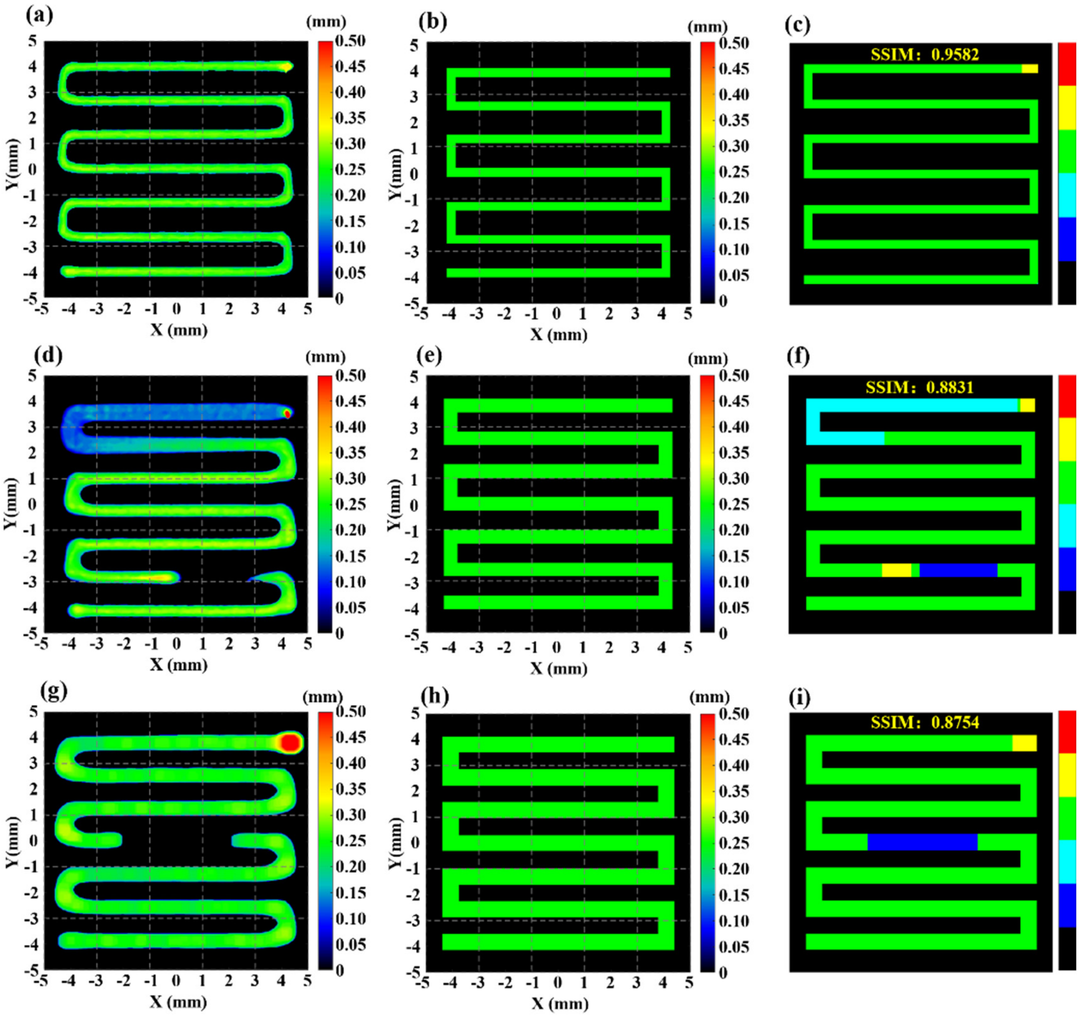

Figure S5. Defect detection results at different printing speeds. (a) Reconstructed model based on 3D P-OCT at higher printing speed (15 mm/s) (b) Target model for (a). (c) Defect characterization map for (a), where red indicates stringing, yellow indicates over-extrusion, green indicates normal extrusion, cyan indicates under-extrusion, dark blue indicates broken filament, and black represents the background. (d) Reconstructed model based on 3D P-OCT at moderate printing speed (10 mm/s). (e) Target model for (d). (f) Defect characterization map for (d). (g) Reconstructed model based on 3D P-OCT at slower printing speed (5 mm/s). (h) Target model for (g). (i) Defect characterization map for (g).

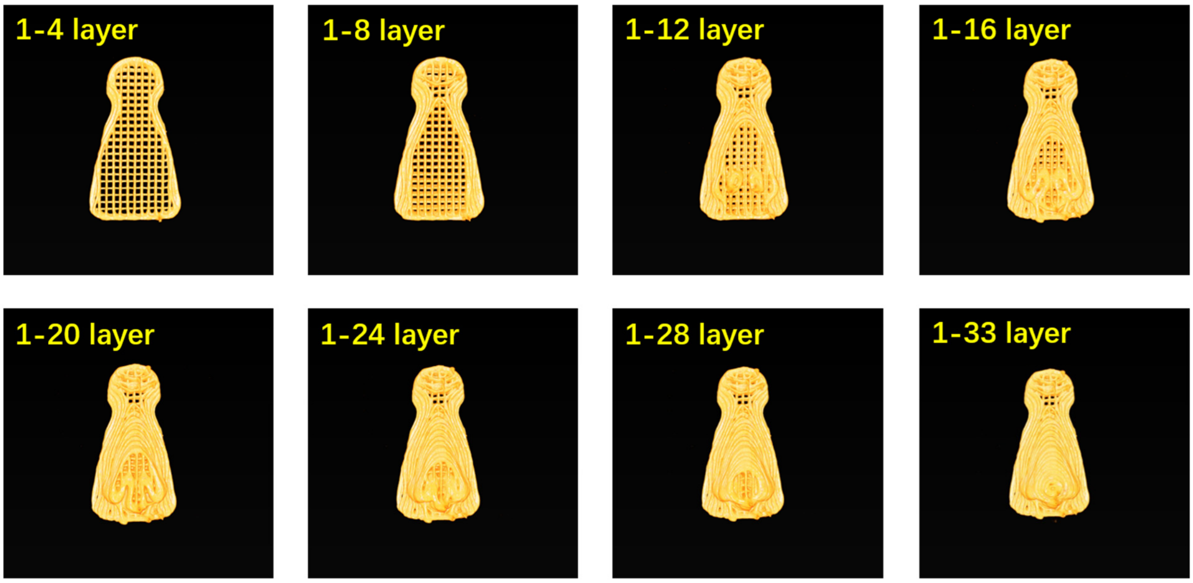

Figure S6. 3D P-OCT reconstruction results of the nose model during the printing process with the alternating "printing-imaging" mode.

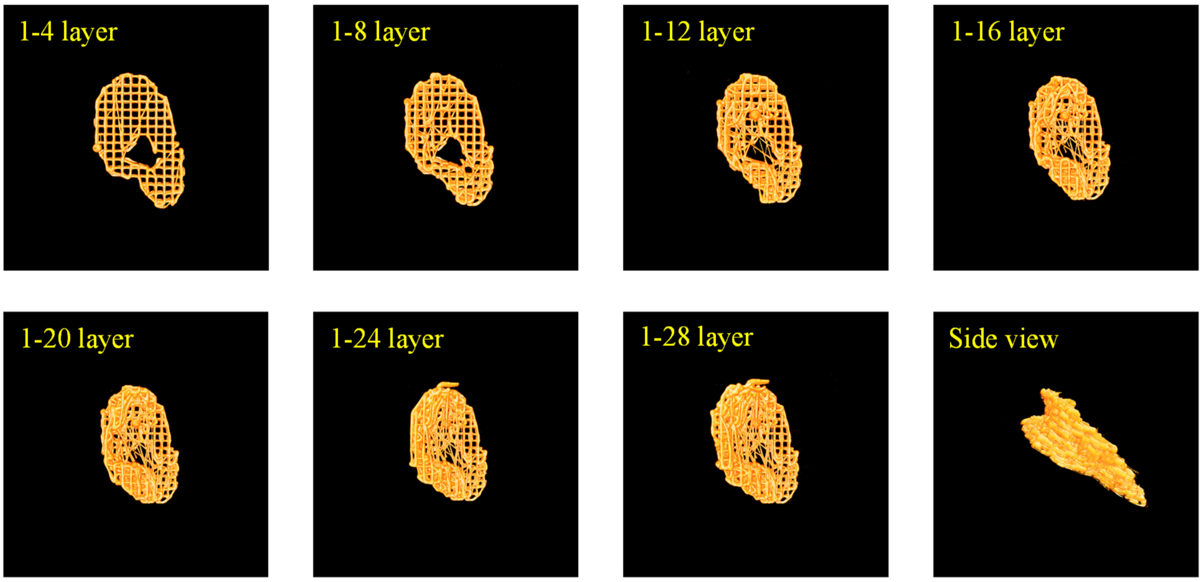

Figure S7. Ear Model with 90-Degree Filling: OCT Rendering Image after Horizontal and Vertical Stitching.

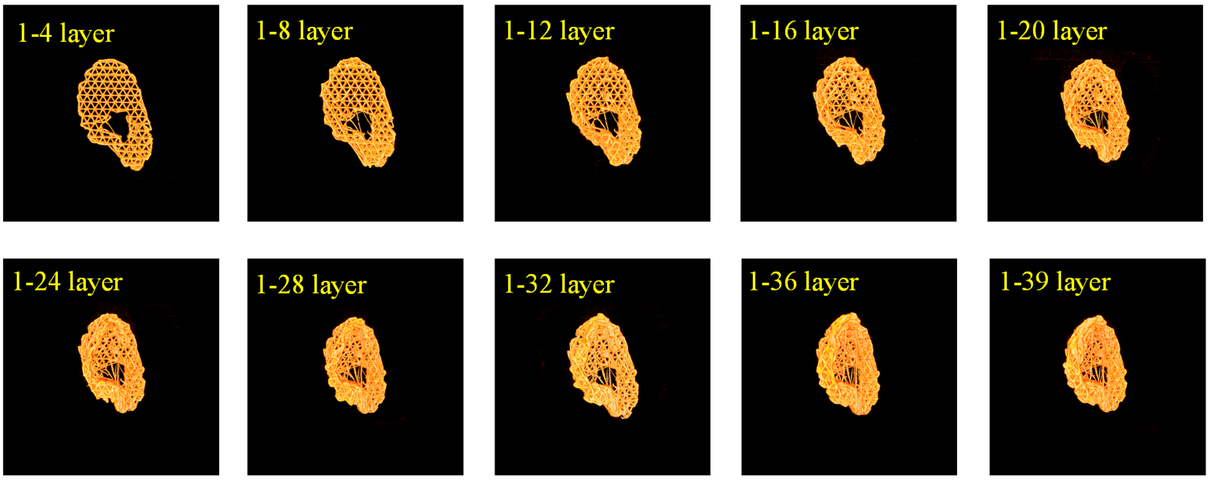

Figure S8. Ear Model with 60-Degree Filling: OCT Rendering Image after Horizontal and Vertical Stitching.

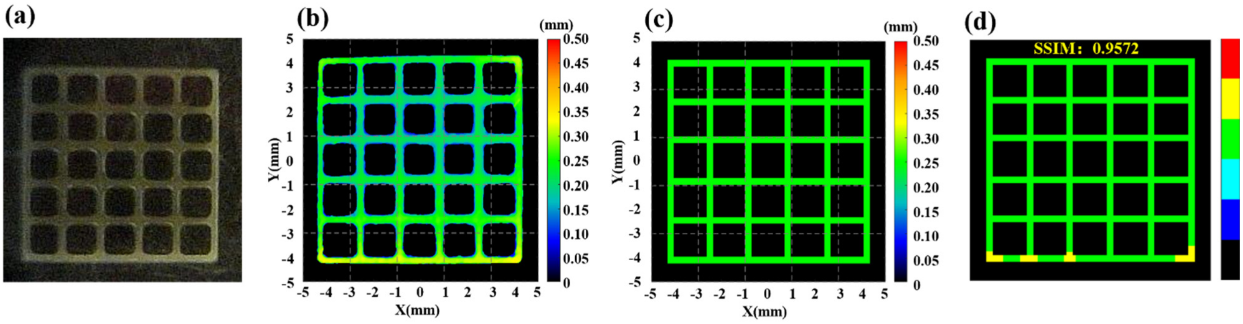

Figure S9. Defect detection for digital light processing (DLP) 3D printing. (a) Lattice grid pattern printed by DLP. (b) 3D P-OCT based reconstruction of the printed model for (a). (c) Target model for (b). (d)Defect characterization map for (b): red indicates stringing, yellow indicates over-extrusion, green indicates normal extrusion, cyan indicates under-extrusion, dark blue indicates filament breakage, and black indicates the background.
